# Ecogenotoxicological studies for an early toxicity screening and monitoring in *Epinephalus chlorostigma* and *Scamberomorus commerson*

**DOI:** 10.1016/j.sjbs.2021.12.064

**Published:** 2022-01-04

**Authors:** Shahid Mahboob, Zubair Ahmed, Muhammad Farooq Khan, Changwei Saho, Promy Virik, N. Al-Mulhm, Almohannad A.A. Baabbad

**Affiliations:** a Department of Zoology, College of Science, King Saud University, Riyadh 11451, Saudi Arabia; b Bioproducts Research Chair, Department of Zoology, College of Science, King Saud University, Saudi Arabia; c Key Lab of Sustainable Development of Marine Fisheries, Ministry of Agriculture, Yellow Sea Fisheries Research Institute, Chinese Academy of Fishery Sciences, Qingdao, China; d Laboratory for Marine Fisheries Science and Food Production Processes, Qingdao National Laboratory for Marine Science and Technology, Qingdao, China

**Keywords:** Comet assay, *Epinephalus chlorostigma*, *Scamberomorus commerson*, Genetic damage, Marine ecosystems

## Abstract

The study was planned to investigate DNA fragmentation in fish to screen aquatic toxicity and in *Epinephalus chlorostigma* and *Scamberomorus commerson* collected from Red sea near Jizan, Saudi Arabia from three locations “(Corniche North park: “16.92161, 42.54631; Jizan Port: 16.874, 42.54952” N and Jizan Economic City: 17.26589, 42.34738“ ”)“ were used as a case study for the application of comet assay. The study area of the Red Sea is polluted due to anthropogenic activities and the disposal of wastes from multiple sources. Comet and micronucleus assays were used to detect genotoxicity in these fish species harvested from three sites. The concentration of Pb, Cr, Zn, Mn, Cu, Cd, Sn, and Hg was higher in the water samples collected from the polluted site compared to the non-polluted site of the Red sea. Comet assay for *S. commerson* showed significant (p < 0.05) genetic damage about 44.33 ± 3.03% DNA in comet tail at site S1. It was subsequently reduced to 31.71 ± 3.52% and 22.11 ± 2.52% at sites S2 and S3. *E. chlorostigma* also showed significant DNA in comet tail as 17.34 ± 2.19%, 11.87 ± 3.01%, and 36.41 ± 3.98% at site S1-S3, respectively. Significant (p < 0.05) DNA damage was observed in the fishes procured from non-polluted locations and upstream locations. The micronucleus induction in *E. chlorostigma* was recorded as 23.20 ± 4.19 and 2.20 ± 0.58%, respectively, non-polluted and polluted sites. *S. commerson* exhibited significant differences between polluted and non-polluted sites (44.80 ± 3.73 and 8.20 ± 2.20‰) polluted and upstream (44.80 ± 3.73 and 20.60 ± 4.02‰), respectively. A significant difference was obtained between *E. chlorostigma* and *S. commerson* for nuclear abnormalities *S. commerson* showed higher frequencies for nuclear deformities than *E. chlorostigma. S. commerson* showed substantial micronucleus induction frequencies collected from an area of low pollution intensity (upstream). This study showed that *E. clorostigma and S. commerson* could be successfully used as a bioindicator to determine the health of the Red Sea through the most specific assays such as comet and micronucleus tests as an early warning and to devise the monitoring strategies to ensure a safe supply of fish for human consumption.

## 1 Introduction

The marine environment has become polluted with an extensive range of toxins, causing worldwide attention over the last few years (Amoozadeh et al., 2014). The petrochemical and oil industry are substantial sources of air contamination; as an outcome of a fast track industrial development daily, which is a source of a massive quantity of industrial discharge into nature without any treatment. This triggers marine fishes, crabs, shrimp, and oysters (Mitra et al., 2010). Substantial, heavy metals in water and sediment pose possible environmental threats and damage human health through trophic transfer (Lu et al., 2011, Komárek et al., 2008).

Water contamination is one of the most distressing issues of humankind. The responsibility for this act goes towards untreated disposal of industrial and domestic discharges into nearby aquatic ecosystems (Claxton et al., 1998, Dixon et al., 2002). Marine fishes could be used as a bioindicator to monitor contamination in the marine ecosystem. Genomic damage caused by genotoxic agents can lead to mutations that require persistent monitoring and detection (Villela et al., 2006). Fishes play an imperative role in the food chains. They are outstanding model bioindicators of the health of aquatic organisms. They can bioaccumulate toxicants directly and indirectly through the food or medium they live (Cavas and Gozukara, 2005; El-Shehawi and Seehy, 2007, Biagini et al., 2009). Such aquatic model animals have an essential role as bioassays to monitor marine water bodies for the intensity of contamination. Toxicants affect the aquatic environment and human health directly or indirectly. Recently research interest in using biomarkers and bioindicators to study the genotoxicity in fishes is developing (Cavas and Gozukara, 2005; El-Shehawi and Seehy, 2007). Fishes respond to various contaminants and genotoxic agents at low concentrations and bioaccumulate them through a heterotrophic web (Goksoyr et al. 1991). The need is to develop molecular biomarkers to mark the effects of environmental contaminants through these bioassays.

Measurement of cytogenetic damage is essential for detecting pollution threats in water bodies (Dixon et al., 2002). Ostling and Johanson (1984) established a microgel electrophoresis technique known as comet assay to detect DNA fragmentation at a single-cell level. This assay requires a small number of cells and is quite sensitive to detecting low genetic damage levels (Tice et al., 2000). Another most reliable and sensitive assay is the micronucleus test. This technique has been proved as a marker for cytogenetic damage caused by clastogenic and aneugenic compounds. This assay was basically developed for mammals, but it has also been applied in fishes (Baršienė et al., 2013). The present study aimed to estimate the genotoxic potential of water pollution in *Epinephalus chlorostigma and Scamberomorus commerson* collected from the Red Sea near Jizzan, Saudi Arabia.

## 2 Materials and methods

### 2.1 Study area

The shoreline of Saudi Arabia has to do with 1840 km in size, representing 79% of the eastern coast of the Red Sea (MEPA/IUCN 1987). The Province of Jizan hinges on the southwest area of the Kingdom of Saudi Arabia. The location remains in standard approximately 50 m water deepness as well as125 kilometers width with coral reefs and low lying sedimentary rock islands. The Jizan location has a subtropical desert environment, and water drainage is primarily westward. Several ephemeral wadi systems drain pipes to the rack, like Jizan, Mais, Bish, and others (Basham, 2009).

Yearly rainfall in this field is more significant than the majority of various other parts of the coastal level; it varies from 50 to 100 mm at the coastline to as high as 500–600 mm inland (Blank et al., 1987) Jizan is a crucial industrial facility, a port and also a facility of farming.

**Fig. 1:**
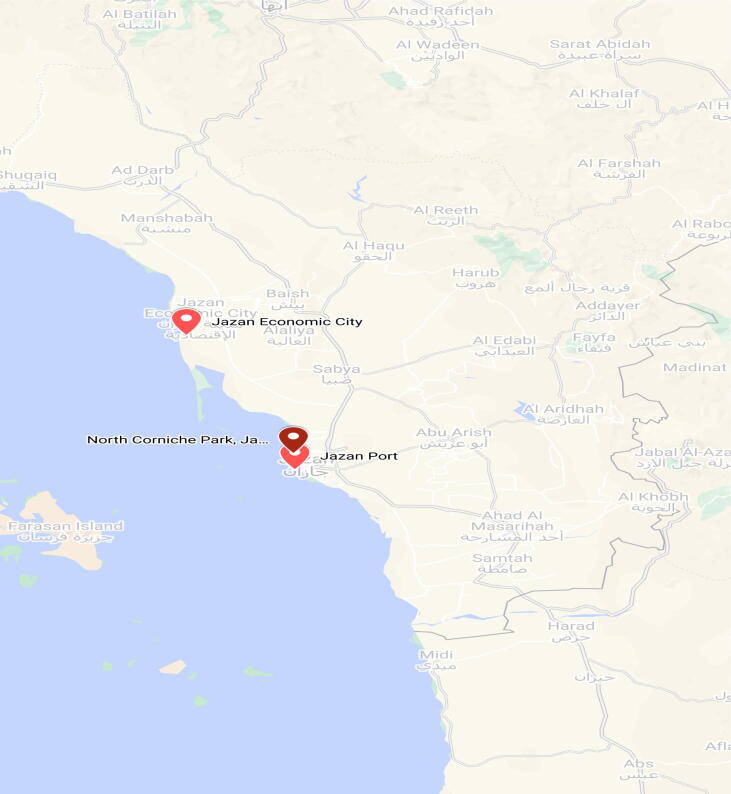
Map of the Study of Jizan, Saudi Arabia, expanding from Corniche North park, Jizan Port and Jizan Economic City.

The present research study focused on the seaside location of Jizan, Saudi Arabia, expanding from Corniche North park: “16.92161, 42.54631; Jizan Port: 16.874, 42.54952“ N and Jizan Economic City: 17.26589, 42.34738” (Fig. 1), to review different contamination sources that affect this location. Three unique sites, specifically Corniche North Park (Location 1: S1), Fish touchdown facility (Location 2: S2), and Sea Port (Location 3: S3), that have business, commercial and farming centers, were included in this research study.

### 2.2 Water sampling and analyses

Polluted water samples were collected in water sampling bottles from selected sites (S1-S3) from the pre-determined stations from the study area. These water samples were analyzed for selected water quality parameters and heavy metals. Five water samples having a volume of 1.5L each were collected and analyzed by the protocol described by Boyd (1981) to meet calculation standards. The concentration of each metal was detected by atomic absorption spectrophotometer (2000 series, H-Tech. Corp. Tokyo, Japan) and metal kits (Spectroquant® Analysis System, Merck).

### 2.3 Fish procurement and blood sampling

*Epinephalus chlorostigma (E. clorostigma) and Scamberomorus commerson (S. commerson*), were collected from the Red Sea near Jizzan, Saudi Arabia. Five specimens from each site (S1, S2, and S3) were collected. Fishes harvested from upstream to this polluted area and non-polluted sites were considered as a control. Fresh fish blood was collected from the caudal vein of each specimen. Blood was used for tests, and the remaining blood was preserved in heparin-coated tubes for four days. The weight of each fish specimen ranged from 1250 to 1500 g.

### 2.4 Comet assay

40 μL of blood was diluted with phosphate buffer saline and stored in ice. This assay was performed on fish RBCs (Singh et al., 1988) with some adjustments in a protocol followed by Cavalcante et al. (2008). Erythrocytes were suspended in low melting point agarose on clean microscopic slides. Slides were immersed in lysing buffer for one hour at 4 °C. This step will help in the unwinding of DNA. Slides were subjected for electrophoresis in electrophoresis buffer (20 min, 300 mA, ∼25 V) and neutralization (three washes of 5 min each in buffer). Ethidium bromide was used to stain slides. Slides were then examined under a fluorescent microscope. Genetic damage was measured in 250 random cells through the software Comet Score V5.

### 2.5 Micronucleus test

Fresh fish blood was smeared on the slides with the help of a coverslip. Slides were air-dried at room temperature, and these slides were fixed in cold Corney fixative for five minutes and left to dry at room temperature. Slides were then stained in aqueous 10% Giemsa stain for 40 min. RBCs' micronuclei induction frequencies were calculated under a binocular microscope at 10x × 60x magnification. For each fish species, five specimens having seven slides each were analyzed. Each fish was analyzed for a total of 35,000 erythrocytes. The frequencies of micronuclei induction were scored according to a protocol adapted by Carrasco et al. (1990) and Cavas and Gozukara (2005).

### 2.6 Statistical analysis

SPSS 9 software was used to calculate means, standard error, and analysis of variance (ANOVA). Duncan's multiple range (DMR) test was used to compare means. p < 0.05 were considered significant. DNA fragmentation through Comet assay was analyzed by TriTek Comet Score™ Freeware 1.6.1.13 by Tritek Corporation.

## 3 Results

**Fig. 2:**
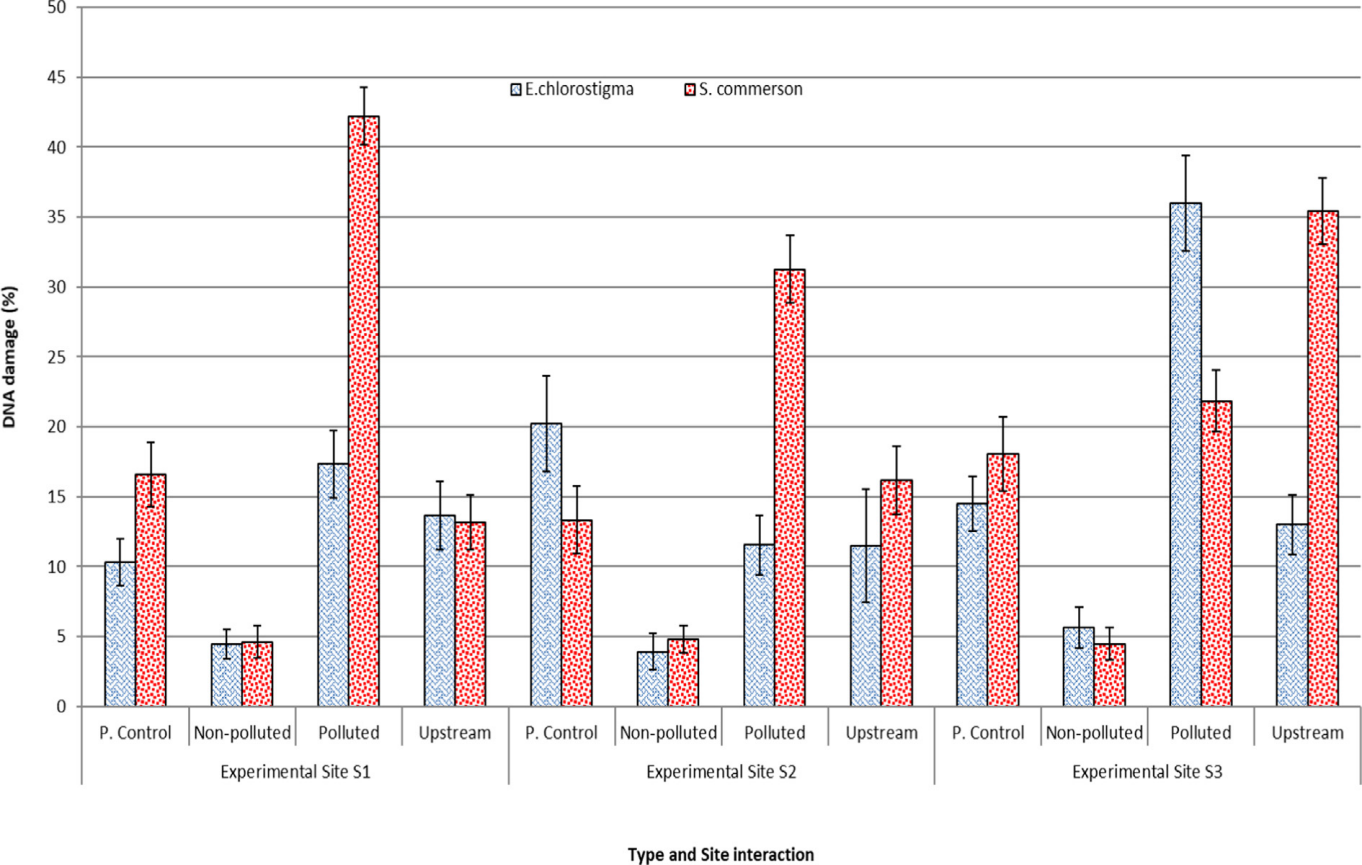
DNA damage in erythrocytes of two fishes *Epinephalus chlorostigma* and *Scamberomorus commerson* collected from three different environments (P. control: Positive control).

**Fig. 3:**
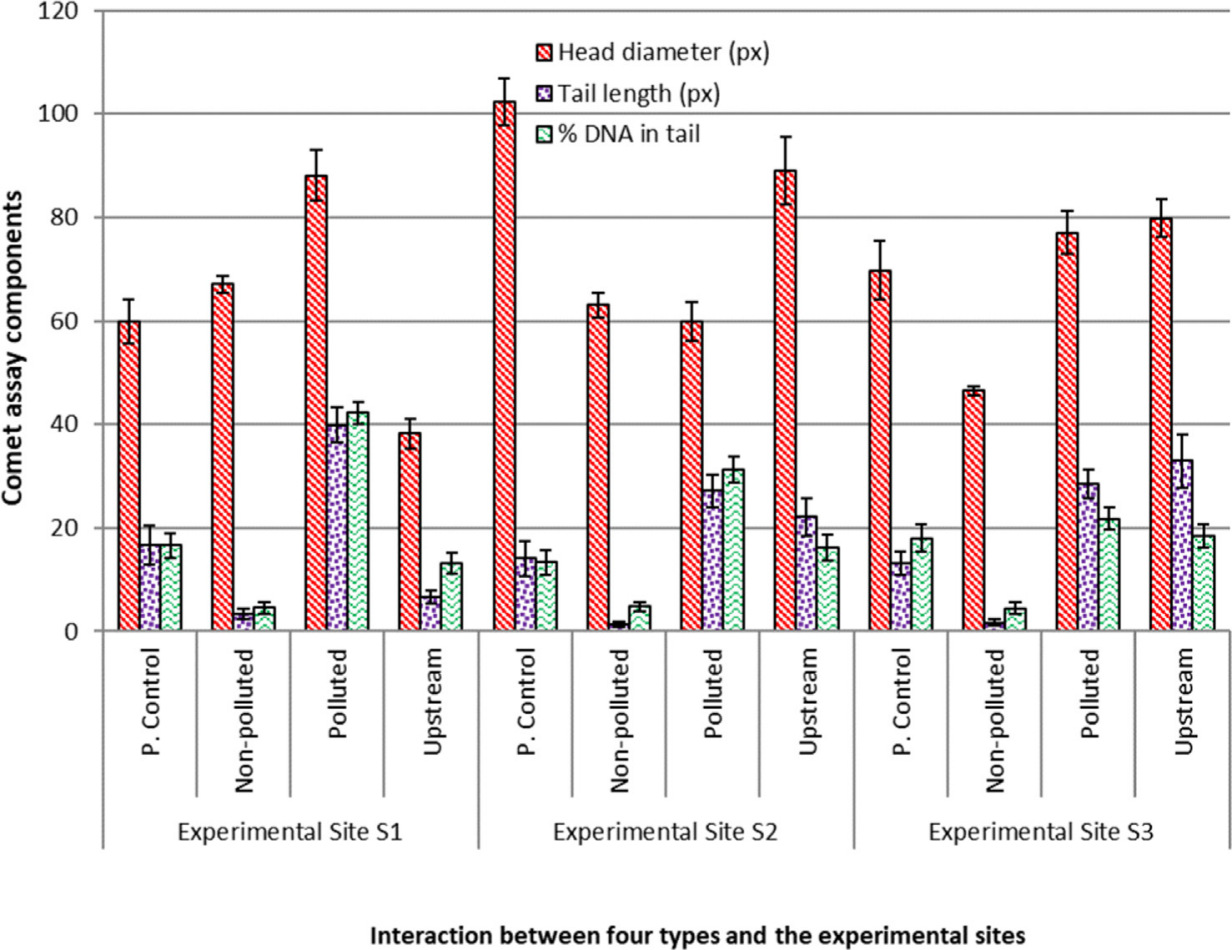
Comparison of three components of comet assays of blood from *Scamberomorus commerson a* collected from three different environments of varying degree of pollution (P. control: Positive control).

*E. clorostigma and S. commerson* were analyzed for genetic damage induced by the pollution in the vicinity of these fishes. All selected heavy metals and water quality parameters were found anomalous and higher enough to make the fish environment unsuitable for living and growing (Table 1). *S. commerson* collected from the polluted areas of the study area showed a significant (p < 0.05) amount of DNA damage (Fig. 2) when compared to the non-polluted site and fish from an area upstream to the polluted area. In the case of *S. commerson* from a non-polluted site, comet length was found to be 64.96 ± 3.22px, comet area 1704.1 ± 53.7px, head diameter 55.94 ± 30.78px, tail length 9.02 ± 12.40px, tail area 233.7 ± 42.85px, DNA in tail 13.19 ± 5.44% (Fig. 3), Tail Moment 1.91 ± 2.78 and Olive Moment 2.06 ± 2.66. In the case of *S. commerson* collected from the polluted area, comet length was found to be 154.36 ± 8.06px, comet area 7818.6 ± 60.3px, head diameter 104.02 ± 32.95px, tail length 50.34 ± 40.38px, tail area 3029.0 ± 63.97px, DNA in tail 37.28 ± 7.77%, Tail Moment 23.48 ± 7.58 and Olive Moment 16.21 ± 14.43. In the case of *S. commerson* collected upstream to the polluted area, the comet length was found to be 212.12 ± 12.1px, comet area 14795.46 ± 75.0px, head diameter 147.32 ± 75.41px, tail length 64.80 ± 44.11px, tail area 2945.1 ± 52.63px, DNA in tail 30.6 ± 2.15%, Tail Moment 23.11 ± 4.90 and Olive Moment 16.40 ± 13.76. Except for tail olive moment, all comet assay components showed significant (p **<** 0.05) differences compared to non-polluted site and polluted area fish categories, while DNA in tail showed highly significant differences. In the case of polluted area and upstream area fish comparison comet height, tail length, tail area, and % DNA in tail showed significant results.

**Table 1:** Water quality parameters of the River Chenab from the points of fish harvest (Mean ± SE).

**Sites**	**Physicochemical parameters (**mgL^-1^) **of water from the Red Sea**											
	Lead			Chromium			Zinc			Manganese		
S1	1.502	±	0.16^C^	0.351	±	0.04^D^	0.215	±	0.04^E^	1.59	±	0.15^C^
S2	1.349	±	0.13^D^	0.288	±	0.05^E^	0.207	±	0.03^F^	1.53	±	0.148^C^
S3	1.299	±	0.13^D^	0.247	±	0.08^F^	0.206	±	0.04^F^	1.36	±	0.14^D^

	Copper			Cadmium			Tin			Mercury		
S1	0.907	±	0.21^E^	0.139	±	0.01^C^	0.305	±	0.06^D^	0.995	±	0.05^BC^
S2	0.863	±	0.13^EF^	0.135	±	0.01^C^	0.275	±	0.04^DE^	1.014	±	0.03^BC^
S3	0.826	±	0.20^F^	0.130	±	0.02^CD^	0.262	±	0.04^E^	0.894	±	0.02^CD^

	pH			Sulfates			Salinity			TDS		
S1	10.37	±	0.05^CD^	264.79	±	28.23^D^	1392.86	±	153.16^E^	1597.64	±	221.95^E^
S2	10.28	±	0.02^D^	250.36	±	47.27^E^	1250.00	±	145.19^F^	1475.43	±	280.16^F^
S3	10.06	±	0.04^E^	246.07	±	45.68^E^	921.43	±	87.15^G^	1214.43	±	237.61^G^

	BOD			COD			Phenols			Conductivity mS/m		
S1	70.64	±	2.33^F^	146.43	±	13.6^F^	1.67	±	0.15^E^	2.25	±	0.26^E^
S2	61.70	±	1.88^G^	135.00	±	13.4^G^	1.48	±	0.10^F^	2.11	±	0.27^F^
S3	50.88	±	1.44^H^	124.07	±	13.9^G^	1.32	±	0.13^G^	1.71	±	0.32^G^

**Fig. 4:**
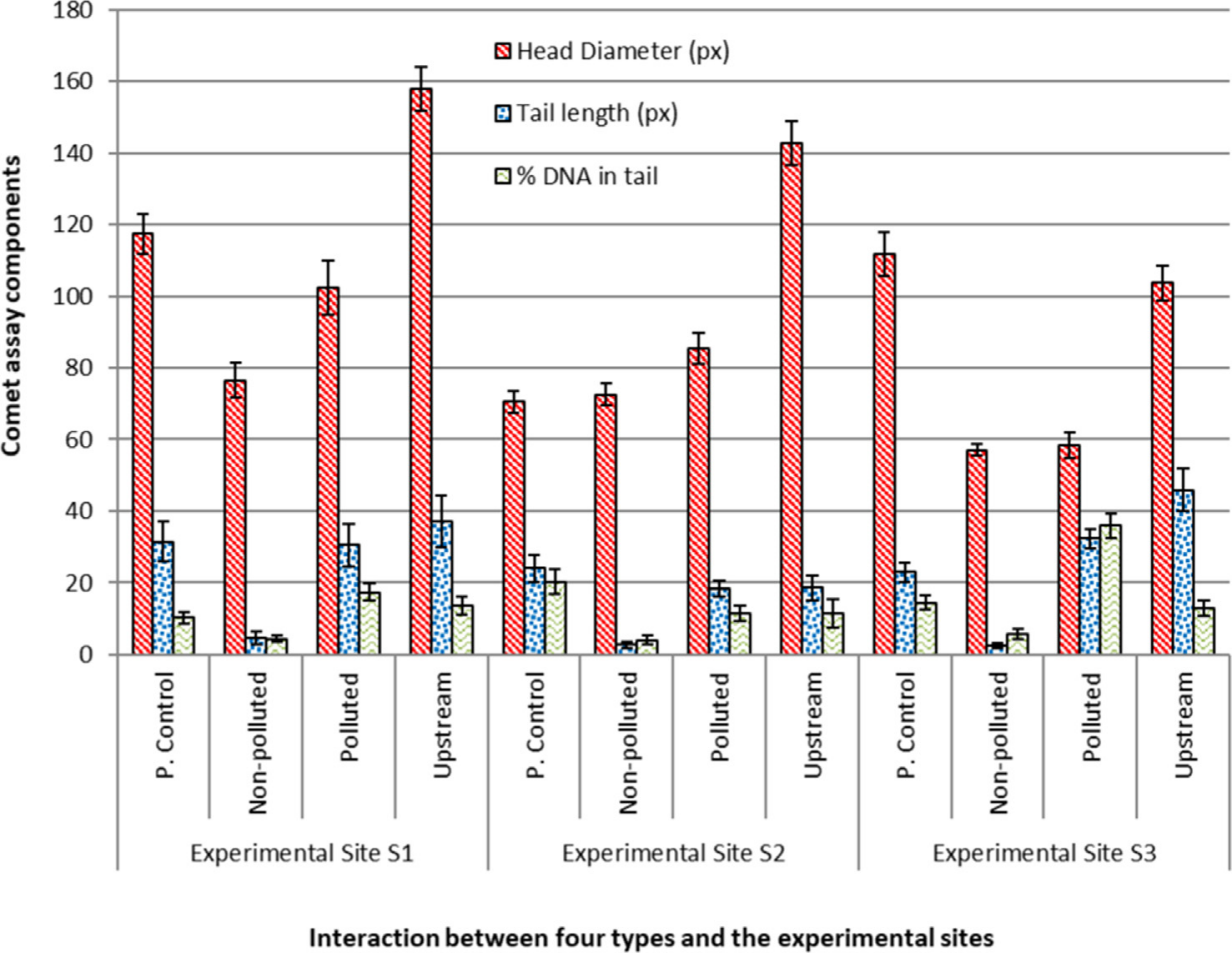
Comparison of three components of comet assays of blood from *Epinephalus chlorostigma* collected from three different environments of varying degree of pollution (P. control: Positive control).

**Fig. 5:**
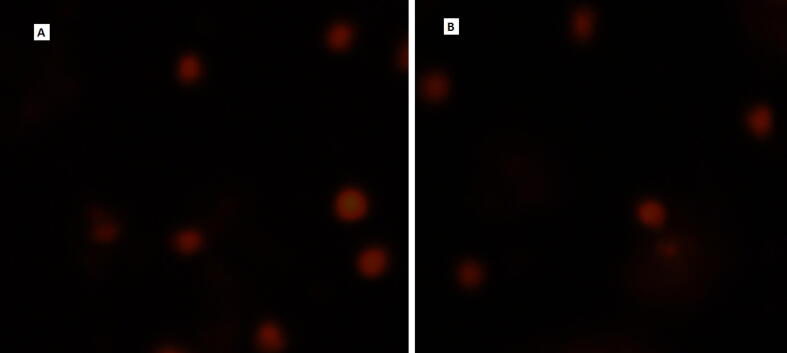
Comet Assay images of two fishes *Epinephalus chlorostigma* (A) and *Scamberomorus commerson* (B).

Means sharing a similar letter in a row or in a column are statistically non-significant (P > 0.05). BOD; Biochemical Oxygen demand, COD; Chemical Oxygen demand. S1-S3; Polluted experimental sites in the River,

In the case of *E. clorostigma* collected upstream to the polluted area, the comet length was found to be 194.98 ± 6.11px, comet area 18530.94 ± 81px, head diameter 157.98 ± 8.2px, tail length 37.00 ± 5.12px, tail area 4287.10 ± 80.8px, DNA in tail 13.66 ± 1.1%, Tail Moment 13.08 ± 2.1 and Olive Moment 12.06 ± 0.63 (Fig. 4). *E. clorostigma* collected from the polluted area the comet length was found to be 132.88 ± 8.35px, comet area 8917.94 ± 66.9px, head diameter 102.42 ± 7.89px, tail length 30.46 ± 4.49px, tail area 2149.70 ± 51.4px, DNA in tail 17.32 ± 3.09%, Tail Moment 10.05 ± 1.13 and Olive Moment 8.85 ± 11.99. *E. clorostigma* collected upstream to the polluted area; the comet length was recorded as 148.92 ± 5.47px, comet area 11776.88 ± 64.3px, head diameter 117.46 ± 9.35px, tail length 31.46 ± 3.77px, tail area 1503.04 ± 58.9px, DNA in tail 10.30 ± 1.95%, Tail Moment 6.30 ± 1.75 and Olive Moment 5.97 ± 1.80 (Table 2). In the *E. clorostigma* comet area, head diameter, tail area, and DNA in comet tail showed significant (p **<** 0.05) differences compared to non-polluted and polluted area fish. In the case of contaminated size and upstream area fish comparison, comet head diameter and % DNA in comet tail showed significant differences (Fig. 5). DNA damage (DNA in the comet tail) for the *S. commerson* showed significant differences compared to the *E. clorostigma*, and *S. commerson* showed more DNA in the comet tail. This greater intensity of genetic damage in *S. commerson* indicates its sensitivity towards pollution.

**Table 2:** Comet Assay analyses in fish species and site interaction.

**Comet parameters**	***S. commerson***				
	Non-Polluted (S1) (Mean ± SD)	Polluted (S2) (Mean ± SD)	Upstream S3 (Mean ± SD)	P (Non-polluted × Polluted)	P (Upstream × Polluted)
Comet Length (px)	64.96 ± 3.22	154.36 ± 8.06	212.12 ± 12.1	**<**0.05	>0.05
Comet Height (px)	57.34 ± 6.82	113.36 ± 5.67	149.18 ± 8.9	**<**0.05	**<**0.05
Comet Area (px)	1704.1 ± 53.7	7818.6 ± 60.3	14795.46 ± 75.0	**<**0.05	<0.01
Head Diameter (px)	55.94 ± 30.78	104.02 ± 32.95	147.32 ± 75.41	**<**0.05	>0.05
Tail Length (px)	9.02 ± 12.40	50.34 ± 40.38	64.80 ± 44.11	**<**0.05	**<**0.05
Tail Area (px)	233.7 ± 42.85	3029.0 ± 63.97	2945.1 ± 52.63	**<**0.05	**<**0.05
% DNA in Tail	13.19 ± 5.44	37.28 ± 7.77	30. 6 ± 2.15	<0.01	**<**0.05
Tail Moment	1.91 ± 2.78	23.48 ± 7.58	23.11 ± 4.90	**<**0.05	>0.05
Olive Moment	2.06 ± 2.66	16.21 ± 14.43	16.40 ± 13.76	>0.05	>0.05

	***E. clorostigma***				
Comet Length (px)	194.98 ± 6.11	132.88 ± 8.35	148.92 ± 5.47	>0.05	>0.05
Comet Height (px)	129.94 ± 8.98	106.16 ± 8.55	110.12 ± 7.58	>0.05	>0.05
Comet Area (px)	18530.94 ± 81	8917.94 ± 66.9	11776.88 ± 64.36	**<**0.05	>0.05
Head Diameter (px)	157.98 ± 8.2	102.42 ± 7.89	117.46 ± 9.35	**<**0.05	**<**0.05
Tail Length (px)	37.00 ± 5.12	30.46 ± 4.49	31.46 ± 3.77	>0.05	>0.05
Tail Area (px)	4287.10 ± 80.8	2149.70 ± 51.4	1503.04 ± 58.9	**<**0.05	>0.05
% DNA in Tail	13.66 ± 1.1	17.32 ± 3.09	10.30 ± 1.95	**<**0.05	<0.01
Tail Moment	13.08 ± 2.1	10.05 ± 1.13	6.30 ± 1.75	>0.05	>0.05
Olive Moment	12.06 ± 0.63	8.85 ± 11.99	5.97 ± 1.80	>0.05	>0.05

P: Probability, Highly significant (P < 0.01), Significant (P < 0.05), Non-significant (P > 0.05).

**Fig. 6:**
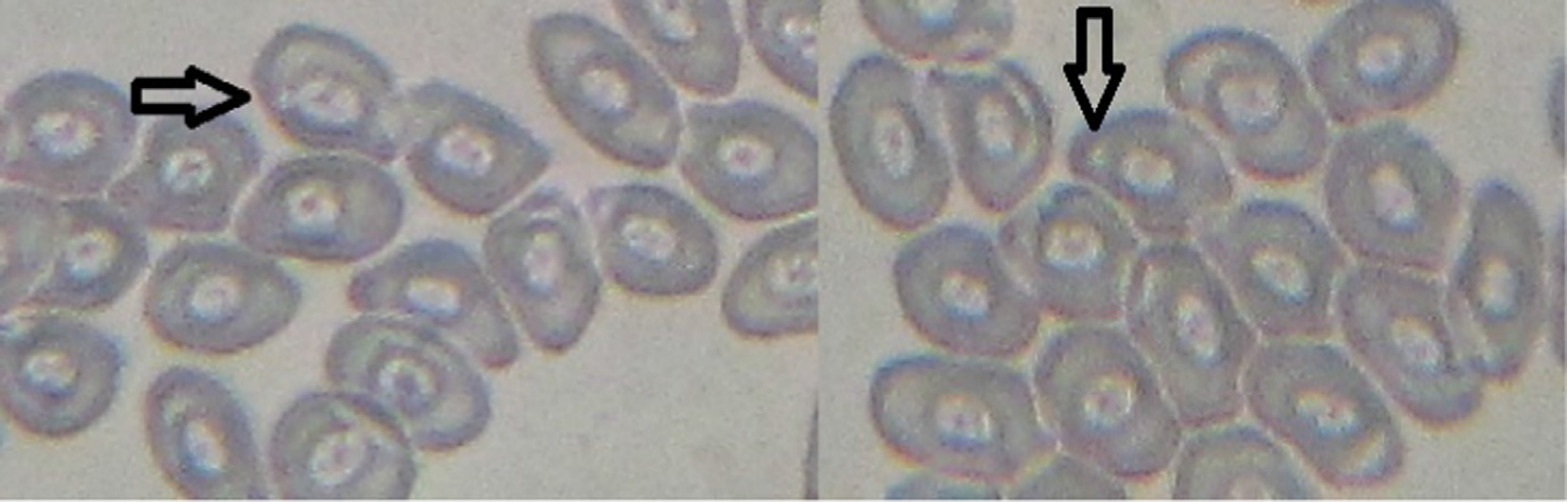
Micronucleus assay of fish (*Epinephalus chlorostigma*) blood harvested from polluted area indicating micronucleus induction (contrast).

**Fig. 7:**
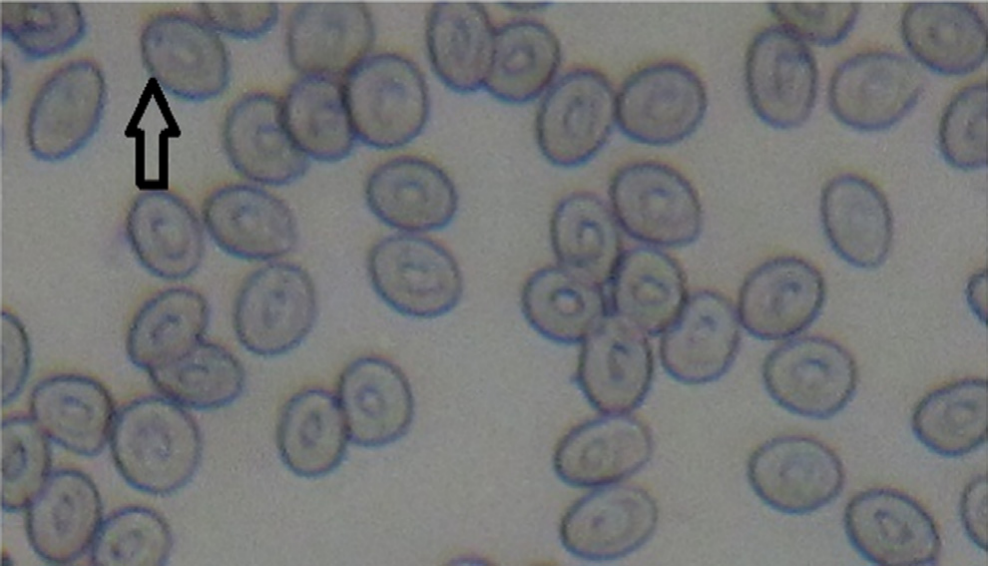
Micronucleus assay of fish (*Scamberomorus commerson*) blood harvested from non-polluted area of the River Chenab indicating low frequency of micronucleus induction.

**Fig. 8:**
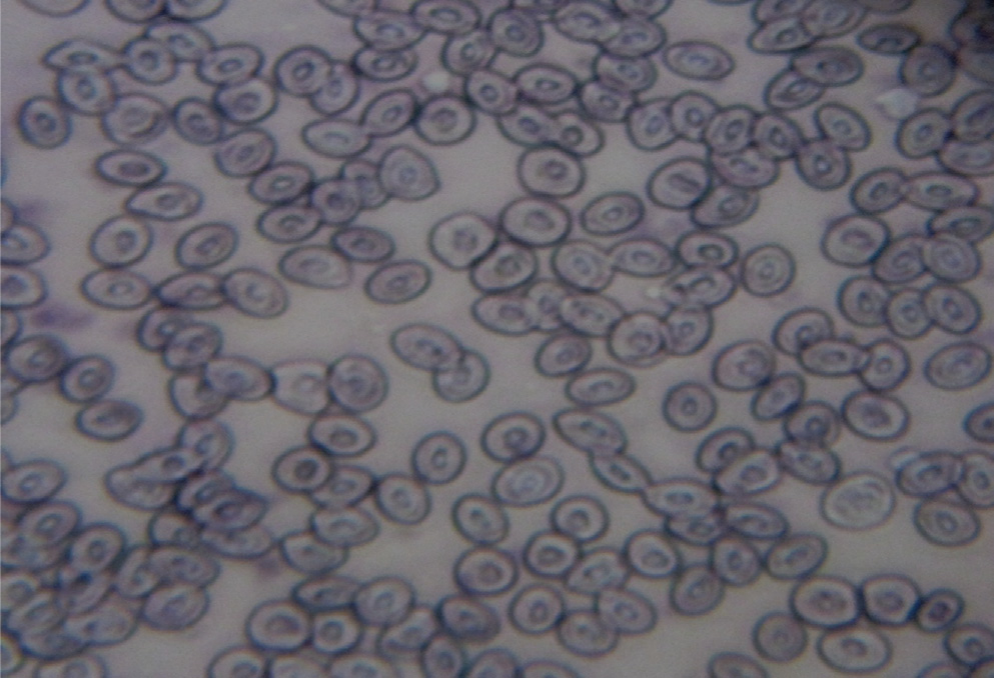
Micronucleus assay of farmed fish (*Scamberomorus commerson*) indicating normal cell structures.

The case of *E. clorostigma* collected from this contaminated area of the Red Sea and non-polluted site showed significant differences of 23.20 ± 4.19 and 2.20 ± 0.58‰ (Fig. 6), but upstream and non-polluted showed non-significant results as 8.0 ± 1.05 and 2.20 ± 0.58‰ (Table 3). Fish from the polluted area and non-polluted site of the Red Sea near Jizan also showed significant differences. *S. commerson* showed significant differences between fish harvested from this highly contaminated area of the river (Fig. 7) and non-polluted (44.80 ± 3.73 and 8.20 ± 2.20‰), polluted and upstream (44.80 ± 3.73 and 20.60 ± 4.02‰), respectively, whereas upstream and non-polluted showed non-significant differences (Fig. 8). In the case of mean comparison for nuclear abnormalities (NA) significant differences were obtained between *E. clorostigma* and *S. commerson*. *S. commerson* showed higher frequencies for nuclear deformities as compared to *E. clorostigma. S. commerson* showed substantial micronucleus induction frequencies collected from an area of low pollution intensity (upstream). It may be because *S. commerson* is a bottom feeder, hence exposed to the polluted sediments.

**Table 3:** Micronucleus inductions (‰) in fish species collected from different sites of varying degree of pollution.

**Type**	**Fish species**			
	***S. commerson***	***E. clorostigma***	***S. commerson***	***E. clorostigma***
	**Single Micronucleus** (‰)		**Double Micronucleus** (‰)	
Polluted	44.8 ± 3.73a	23.2 ± 4.19bc	6.2 ± 0.97ab	2.81 ± 1.07b
Upstream	20.6 ± 4.02bcd	8.0 ± 1.05 cd	5.22 ± 1.53b	1.4 ± 0.75b
Control	8.2 ± 2.20 cd	2.2 ± 0.58d	0.8 ± 0.37b	0.0 ± 0.0b
+ve Control	37.4 ± 3.92ab	43.6 ± 5.35a	8.4 ± 2.80ab	8.6 ± 3.67ab
Mean	27.75 ± 3.66A	19.25 ± 4.00B	5.15 ± 1.0AB	3.20 ± 1.17B

Readings sharing similar letters in a column or row differ statistically non-significantly (P > 0.05). Frequency calculated in thousand cells.

## 4 Discussions:

Urban and industrial untreated sewage discharges are mainly responsible for the contamination of aquatic ecosystems (Claxton et al., 1998, Richards et al., 2000). Information about the genotoxic effects of contaminants on fishes is scanty in literature (Galindo et al., 2010, Pavlica et al., 2011). Contaminants in these ecosystems stimulate genetic alterations (Russo et al. 2004). Present study findings corroborate the study of van der Oost et al. (2003) by using biomarkers in fish as indices of water pollution. Another study reported by Richards et al., 2000, Pietripiana et al., 2002 indicated higher micronuclei frequencies in RBCs of the fish harvested from areas duly contaminated with heavy metals corroborate the results present study. Baršienė et al. (2013) also compared non-polluted and polluted area fish species genotoxic damage and found an increased level of genetic damage in contaminated area fish. Present findings from comet assay data of these fish species agree with the conclusions from Pavlica et al. (2011) for genotoxicity in fish and its use for environmental screening and biomonitoring. As a result of an increase in marine contamination, aquatic fauna and flora have been significantly exposed to adverse effects of harmful contaminants, cancer-causing, and mutagenic agents. In environmental surveillance evaluation, MN assaying has become an easy, low cost and fast technique for evaluating genotoxic effects as evidently, the activity of any genotoxic agent might trigger an increase in MN frequency (Santana et al., 2020). In Turkey, 5 different fish species were harvested from “Aliaga Bay and observed 23–53.33 ‰ MN and 4–32.7 ‰ ”NA in the fish sample collected from contaminated vicinity, contrasted to 11–18 ‰ MN and 1–5 ‰ NA in the specimen collected from the non-polluted area (Arslan et al., 2015, Hussain et al., 2018). Carrasco et al. (1990) reported non- significant differences between NA rates in fish from a contaminated and non-polluted location in the fish samples procured from Ceará estuaries. Whereas, few researchers argued that low rates of MN and NA in fish from the polluted area might be because of adjustment in the degraded environment (Bombail et al., 2001, Seriani et al., 2013) and variables like interspecies level of sensitivity, metabolic capability, DNA repair, protection mechanism (Rodriguez-Cea et al., 2003) and countervailing system because of persistent chemical disruption by a complex blend of hazardous chemicals gradually accumulated into the water and aquatic fauna and flora of such environments (Katsumiti et al. 2009).

The present results confirm the previous studies that showed higher micronucleus induction frequencies in fish species harvested in contaminated waters (Bombail et al., 2001, Pietripiana et al., 2002, de Andrade et al., 2004; Cavas and Gozukara, 2005; Kumar et al. 2010). A laboratory study on fish exposed to textile mill effluents, cypermethrin, and cyclophosphamide showed higher intensity of micronucleus induction in the gills. This study also insisted on using cells from gill or blood erythrocytes for genotoxic studies (Cavas and Gozukara 2003). The use of connective tissues has the advantage of a high mitotic index to indicate genotoxicity (Cavas and Gozukara 2005). All studied water quality parameters were found to be sufficiently higher than WHO permissible limits, showing higher pollution intensities in the fish environment. A study by Viarengo et al., 2007, Pulkrabová et al., 2007 also illustrated that bottom-dweller species suffer more due to water contamination. These findings verify the genotoxic potential of these fish species to be used as a bioindicator of water pollution in the Red Sea, near Jizzan, Saudi Arabia.

## 5 Conclusion

The Red Sea near the port of Jizzan and the economic City area acted as a sink for genotoxicants and was extremely contaminated due to industrial and domestic sewage waste disposal. This study showed that *E. clorostigma and S. commerson* could be successfully used as a bioindicator to determine the health of the Red Sea through the most specific assays such as comet and micronucleus tests as an early warning and to devise the monitoring strategies to ensure a safe supply of fish for human consumption. These findings verify the genotoxic potential of these fish species to be used as a bioindicator of water pollution in the Red Sea, near Jizzan, Saudi Arabia.

## Declaration of Competing Interest

The authors declare that they have no known competing financial interests or personal relationships that could have appeared to influence the work reported in this paper.
